# Axillary artery transection and bilateral pulmonary embolism after anterior shoulder dislocation: case report

**DOI:** 10.1051/sicotj/2016041

**Published:** 2017-01-11

**Authors:** Betty Leclerc, François Loisel, Maxime Ferrier, Mazen Al Sayed, Simon Rinckenbach, Laurent Obert

**Affiliations:** 1 Vascular Surgery Unit, University Hospital of Besançon 25030 Besançon France; 2 Orthopaedic, Traumatology and Hand Surgery Unit, University Hospital of Besançon 25030 Besançon France

**Keywords:** Axillary injury, Bilateral pulmonary embolism, Anterior shoulder dislocation, Neurovascular complications

## Abstract

*Introduction*: Anterior shoulder dislocation can be associated with vascular and neurological complications. However, axillary artery injury associated with shoulder dislocation is rare and extremely rare without bone fracture. An early diagnosis of these complications allows predicting long-term functional outcomes.

*Methods*: This article reports the case of a 66-year-old patient who presented an anterior shoulder dislocation after a ski fall without any neurological dysfunction or pulse deficit.

*Results*: The first reduction attempts were unsuccessful and during the new attempt, we observed a hematoma. A CT scan showed a disruption of the axillary artery and a bilateral pulmonary embolism.

*Conclusion*: Neurovascular injury must be systematically sought before and after reduction, and a multidisciplinary approach is always necessary.

## Introduction

Dislocations of the shoulder are the most common joint dislocations seen in the emergency department. Complications of shoulder dislocations must be recognized early to prevent a long-term impact on the patient functional outcome. Arterial lesions occur in less than 1% of shoulder dislocations. The diagnosis of vascular injuries is based on pain, expanded hematoma, peripheral cyanosis, coolness, pallor, pulse deficit, neurological dysfunction, and possibly a shock. Axillary artery injury associated with shoulder dislocation is rare and extremely rare without bone fracture [1, 2]. The axillary artery is divided into three parts. The first part follows the subclavian artery, begins at the lateral border of the first rib, and extends to the superior border of the pectoralis minor. The second part lies deep in the pectoralis minor muscle. The third part begins at the inferior border of pectoralis minor and at the inferior border of the teres minor muscle. Possible arterial injuries can be an artery occlusion, a rupture, or a pseudoaneurysm. In addition, venous injuries can occur such as subclavian vein thrombosis. To improve the management of such vascular problems, it is necessary to establish an early diagnosis, to use an occlusion balloon to control bleeding, and to perform a surgical treatment. A CT (computed tomography) scan or an angiography must be performed before or during the surgical management. This article reports the case of an axillary artery transection with upper limb ischemia associated with bilateral pulmonary embolism after anterior shoulder dislocation without bone fracture.

## Case report

**Figure 1.**
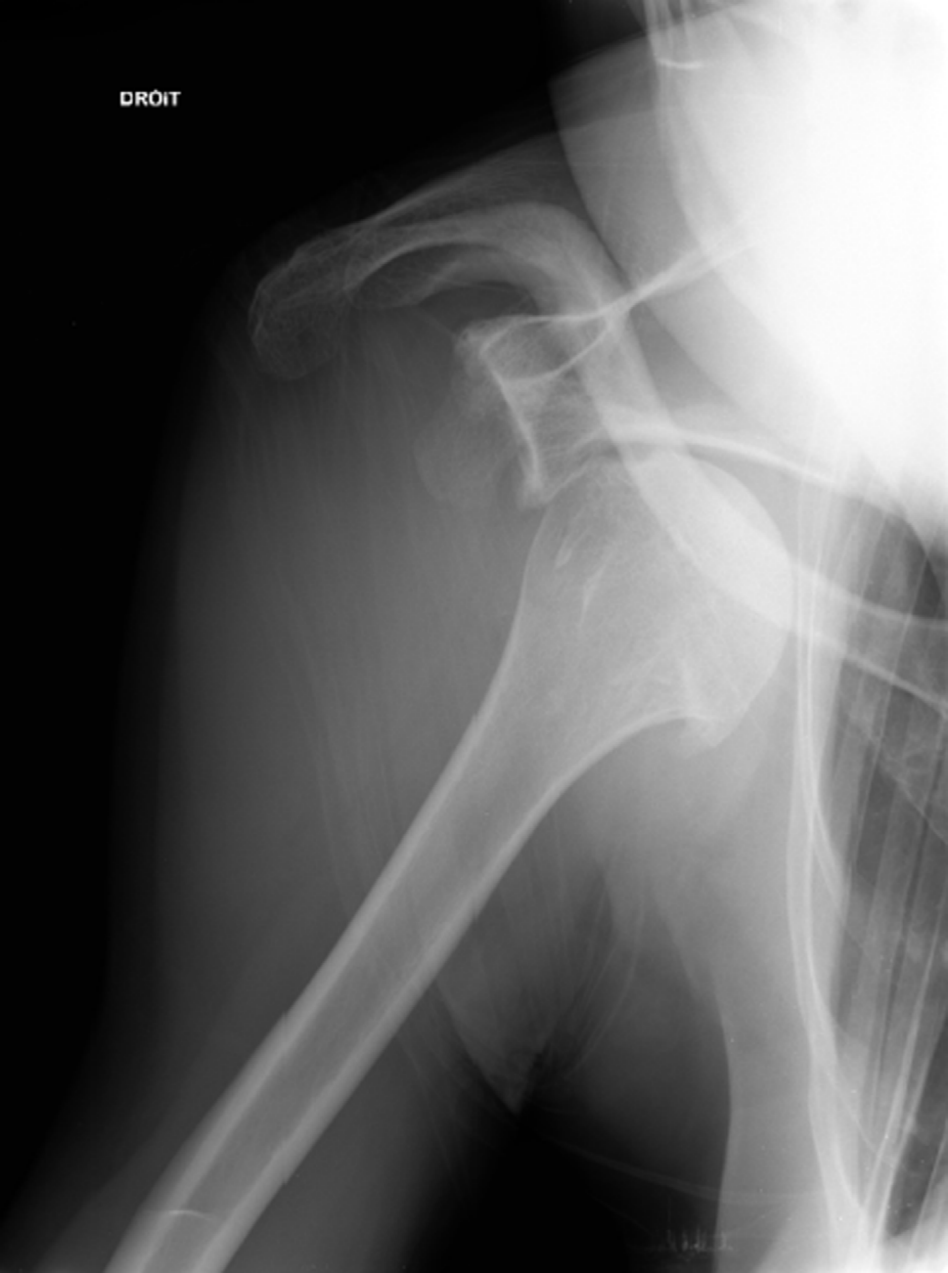
Anterior shoulder luxation.

**Figure 2.**
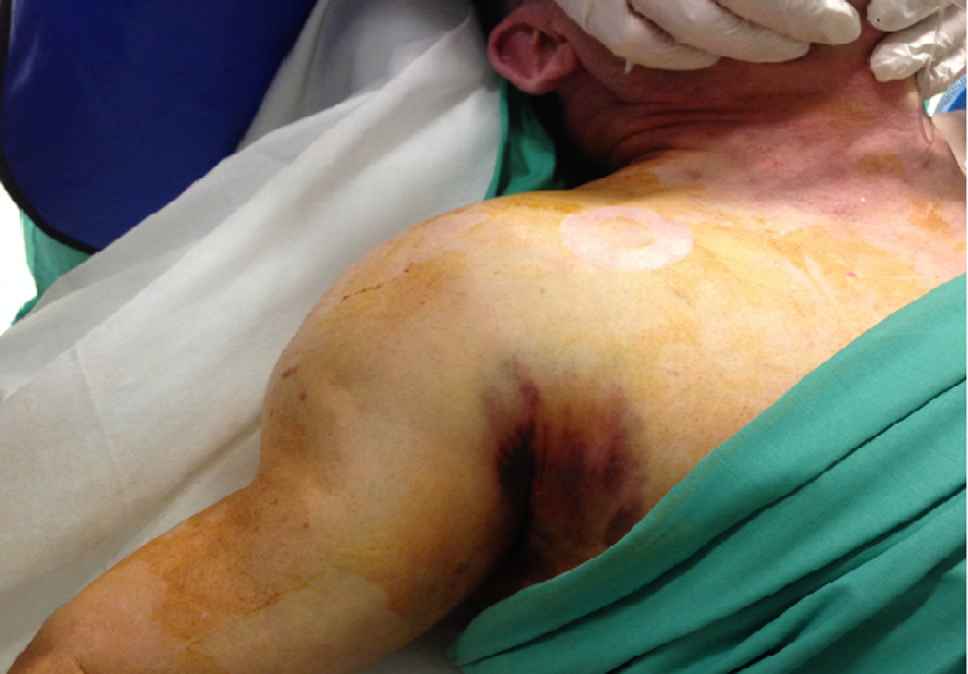
Deformation of the shoulder and hematoma in the deltopectoral region.

**Figure 3.**
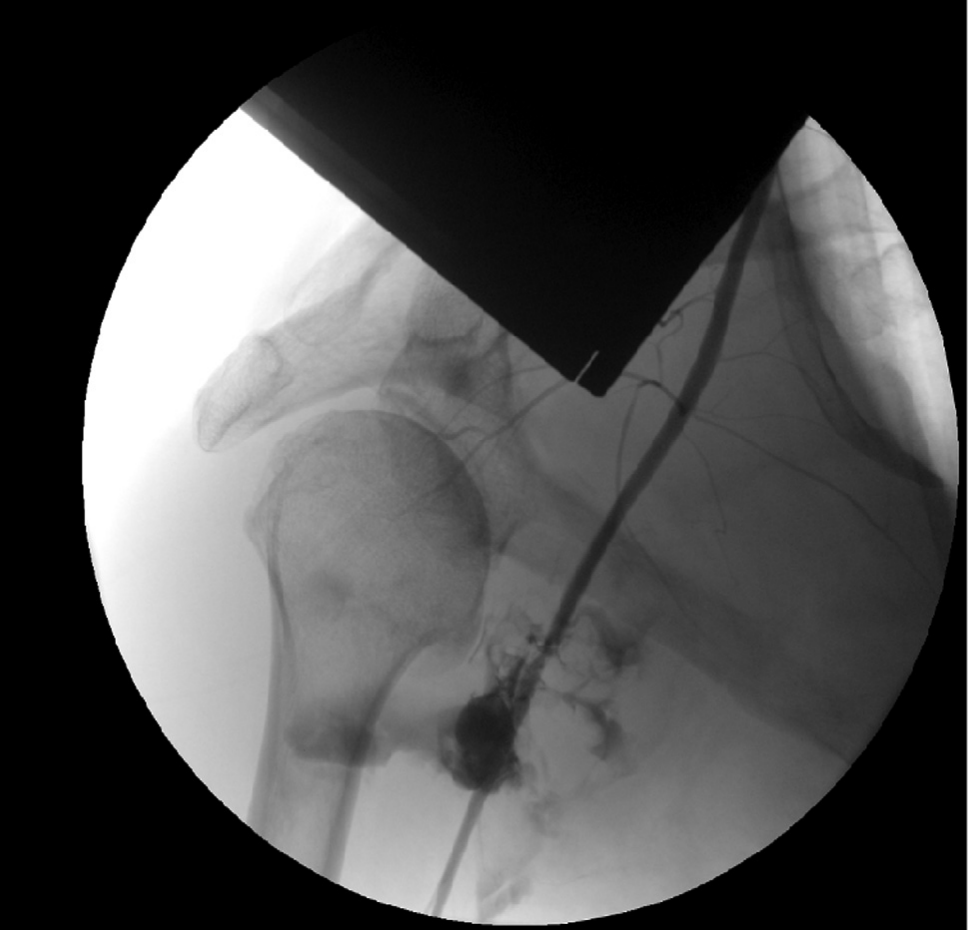
Disruption of the axillary artery on arteriography.

A 66-year-old man presented to emergency department with an anterior shoulder dislocation (Figure 1) after a ski fall without loss of consciousness. At the initial management, the patient did not show any neurological dysfunction or pulse deficit. The first reduction attempts were unsuccessful and the patient was put under general anesthesia before trying again. During the new attempt to reduce the shoulder dislocation, we observed the occurrence of a hematoma in the deltopectoral region (Figure 2). At the same time, loss of palpable peripheral pulse and upper limb ischemia appeared. A CT scan was performed and showed a compressive hematoma in the axillary region, a disruption of the axillary artery flow, and a bilateral pulmonary embolism (previously unknown). The patient was transferred to a university hospital to be supported by vascular and trauma surgeons. The patient was still under general anesthesia. The dislocation was successfully reduced in the operating room. Upper limb ischemia with persistent pulseless and cold arm led to an emergency vascular management. The humeral artery was dissected through the brachial tunnel and a thromboembolectomy was done. An arteriography was performed due to the persistence of pulsatile hematoma, which revealed a disruption of the axillary artery with extravasation of contrast near the humeral head (Figure 3). An angioplasty balloon was introduced to control the proximal bleeding. After the introduction of the occlusion balloon, an incision was performed at the pectoralis major muscle and the hematoma was decompressed. The artery was deteriorated over about 1 cm and an end-to-end anastomosis was performed. The bilateral pulmonary embolism was probably due to the compression of the axillary vein during the shoulder dislocation. Nevertheless, no examination has confirmed this hypothesis. The patient received an intravenous anticoagulant therapy by heparin and then by an oral anticoagulant. Postoperatively, Doppler ultrasound showed a good morphologic and hemodynamic result. Concerning the functional outcome, the patient did not have neurological or vascular complications in the postoperative period. Then he presented with a partial functional disability probably due to a distal brachial plexus stunning according to results of electromyogram. The CT scan showed the absence of significant lesion of the rotator cuff.

## Discussion

Ergünes et al. [2] have published a review of the literature about axillary artery injury after shoulder dislocation. They have shown that the artery transection was seen mainly in the third part of the axillary artery. Vascular reconstruction, such as end-to-end anastomosis, reversed saphenous vein, prosthetic graft, or rarely ligation of the artery, is generally performed. It is necessary to explore the arterial status of the upper limb when a patient has an anterior shoulder dislocation with signs of arterial injury. Conventional angiography is the gold standard and it allows clamping with an occlusion balloon to control proximal bleeding and possible endovascular procedures but CT angiographies are now easily achieved.

An endovascular approach should also be discussed. Indeed, this procedure is less invasive and can be performed under local anesthesia. Brachial plexus injury is also associated in 60% of subclavian and axillary arterial injuries. The neurological deficit is the determinant of a long-term disability.

Menendez et al. described several factors associated with lesions of the axillary artery in patients with a proximal humerus fracture. Male sex and atherosclerosis were independent predictors of axillary artery injury (OR: 1.6, 95% CI 1.2–2.0; *p* < 0.001 and OR: 3.7, 95% CI 2.5–5.4; *p* < 0.001). We propose that the loss of arterial elasticity observed in atherosclerosis makes the axillary artery more susceptible to injury. The authors found that increasing age was not a risk factor for axillary arterial lesion [3]. However in the literature, atherosclerosis is not mentioned as such a risk factor in shoulder dislocation.

There are only few works reporting a deep venous thrombosis or a pulmonary embolism in this context. Venous thromboembolic events are sometimes seen in the inferior shoulder dislocation or in posterior sternoclavicular dislocation [4].

Fracture dislocation of the proximal humerus can sometimes be a source of rare complications. In the literature, there are cases of dyspnea associated with intra-thoracic migration of the humeral head [5].

Neurovascular injury must be systematically sought before and after reduction of the shoulder dislocation. If some clinical sign of an arterial injury is present, a CT angiography or an angiogram must be performed. An early multidisciplinary approach could allow to obtain a better functional outcome.

## Conflict of interest

All authors state that they have no conflict of interest.

## References

[1] Kelley SP, Hinsche AF, Hossain JFM (2004) Axillary artery transection following anterior shoulder dislocation: classical presentation and current concepts. Injury 35(11), 1128–1132. 15488503 10.1016/j.injury.2003.08.009

[2] Ergüneş K, Yazman S, Yetkin U, Cakır V, Gurbuz A (2013) Axillary artery transection after shoulder dislocation. Ann Vasc Surg 27(7), 974.e7–974.e10. 10.1016/j.avsg.2013.04.002 23849653

[3] Menendez ME, Ring D, Heng M (2015) Proximal humerus fracture with injury to the axillary artery: a population-based study. Injury 46(7), 1367–1371. 25986664 10.1016/j.injury.2015.04.026

[4] Chotai PN, Ebraheim NA (2012) Posterior sternoclavicular dislocation presenting with upper-extremity deep vein thrombosis. Orthopedics 35(10), e1542–e1547. 23027495 10.3928/01477447-20120919-27

[5] Joshi SP, Challawar NS, Agrawal PV, Gajjar AS (2016) Dyspnea in a case of shoulder dislocation – to beware of this rare life-threatening symptom. SICOT-J 2, 30. 27658507 10.1051/sicotj/2016022 PMC5034681

